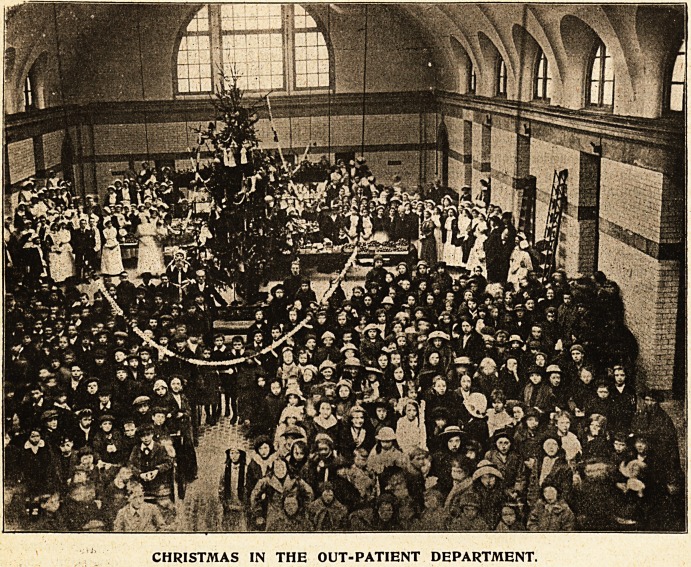# Manchester Royal Infirmary

**Published:** 1917-01-13

**Authors:** 


					300 THE HOSPITAL January 13, 1917.
MANCHESTER ROYAL INFIRMARY.
I
. 3
CHRISTMAS IN THE OUT-PATIENT DEPARTMENT.

				

## Figures and Tables

**Figure f1:**